# Insecticidal effects of nano-encapsulated lemongrass essential oil on the population parameters of *Spodoptera frugiperda* using two-sex life table

**DOI:** 10.1038/s41598-025-93779-8

**Published:** 2025-04-01

**Authors:** Samar Sayed Ibrahim, Magdy Youssef El-Kholy, Shehata El-Sayed Mohamed Shalaby

**Affiliations:** Pests and Plant Protection Department, National Research Centre, 33 El-Buhouth Street, Dokki, 12622 Giza Egypt

**Keywords:** Entomology, Nanoparticles

## Abstract

Essential oils derived from plants have demonstrated their efficacy in controlling insects; however, their poor physical characteristics restrict their widespread application. In this study, nano-encapsulation employing emulsification followed by ultrasonication technique has improved the insecticidal efficacy of lemongrass oil (LGO) against *Spodoptera frugiperda*. Beta-cyclodextrin (β-CD) and gum Arabic (GA) were used as coating materials to protect the vital components of LGO. Different concentrations of LGO pure and nano-capsule formulations have been used to examine the biological development of *S. frugiperda* 1st larval instar using two sex life table. D-Limonene, E-Citral, and Z-Citral were identified as the main compounds in LGO by GC–MS analysis. The oil concentration affected the physical properties of nano-capsules. The LGO nano-capsules had particle sizes ranging from 279.63 to 471.93 nm and an encapsulation efficiency between 51.94 and 83.59%. Following 96 h, the LGO:β-CD/GA treatment of *S. frugiperda* had a lower LC_50_ value (1.52%) than the LGO treatment (2.48%), indicating that the nano-capsules were more toxic. Nano-capsules had a significant adverse effect on the life table parameters of *S. frugiperda*. The results of this study indicate that encapsulating LGO with β-CD/GA inclusion enhanced the efficacy of lemongrass oil against *S. frugiperda*.

## Introduction

The highly destructive pest fall armyworm (FAW) *Spodoptera frugiperda* (J.E. Smith) (Lepidoptera: Noctuidae) attacks maize, rice, and sorghum in addition to other crops belonging to several families^1^. During its larval stage, the polyphagous pest *S. frugiperda* can severely damage plants. The pest made its African appearance in 2016 when it was first detected in Nigeria. It quickly spread to other countries in Southern and Eastern Africa, severely damaging maize crops—with losses topping 70%—as well as sorghum and other crops to a lesser degree^2^. The *S. frugiperda* pest was then identified for the first time in May 2019 in maize-growing areas in Aswan, Egypt^3^. The *S. frugiperda* invasion has put over 200 million Africans at risk of food insecurity. These people’s main staple food is maize, which is also a highly vulnerable crop in all attacked areas^4^. To improve crop yields, pest management uses about 2 million tonnes of chemical pesticides annually, and of these, 29.5% are for insect pest control^3^. Natural plant products are increasingly being used as more sustainable and environmentally friendly alternatives to chemical insecticides. Because essential oils (EOs) have contact, fumigant, attractant, and repellent properties against a variety of insect pests, they could serve as a supplier of distinctive insecticides^5^. Moreover, essential oil from lemongrass (LGO) (*Cymbopogon citratus* (DC.) Stapf., Poaceae) exhibited insecticidal effects against *S. frugiperda*, *Sesamia cretica*, and *S. exigua*^6–8^. However, when applied broadly, essential oil’s high volatility, low solubility, and instability in a variety of environmental conditions prevent it from performing effectively as a bio-insecticide.

The utilization of nano-encapsulation technology could help in resolving these issues. Nano-encapsulation is a technology that encapsulates active substances and produces highly efficient nano-capsules with a greater surface area^9^. By offering high stability and controlled release features, nano-capsules can increase the effectiveness of essential oils^10^, and thus improve their insecticidal properties. Additionally, there could be several advantages to utilizing nano-encapsulated insecticides, including increased toxicity and bioavailability as well as a reduction in the amount of active ingredients required^11^. Adequate wall materials should be employed for effective nano-encapsulation; these materials need to be selected based on a variety of factors, including cost, availability, safety, and biocompatibility. Cyclodextrins are suitable for the encapsulation process because they can form inclusions in an aqueous solution with hydrophobic and organic guest components^12^. Beta-cyclodextrin (ß-CD) is a naturally occurring cyclic oligosaccharide that is a highly effective monoterpene carrier^13^. In addition to having low viscosity at high concentrations, gum Arabic (GA) (Acacia gum) also possesses outstanding characteristics like emulsification, the capacity to form films, and small particle size^14^. Previous studies demonstrated the utilization of ß-CD and/or GA to encapsulate different essential oils such as *Oliveria decumbens*, basil, and oregano oils^15,16^. The Food and Drug Administration (FDA) has classified both LGO and β-CD as generally recognized as safe (GRAS) due to their nontoxicity and environmental friendliness^17,18^.

Studying life table parameters allows for the most effective use of insecticides. According to previous findings, essential oils may have insecticidal toxic effects that include accelerated or delayed development, altered life-table parameters, immature stage mortality, and adult wing deformities^19^. Understanding how lethal concentration affects *S. frugiperda*'s development will improve the knowledge of its control method^20^. Very few studies have been conducted to evaluate the toxicity of nano-formulated essential oils on the life table parameters of insect pests, particularly *S. frugiperda*. Thus, the purpose of this work was to synthesize and characterize LGO in nano-capsule formulations using β-CD: GA inclusion as protective wall material. In addition to evaluate and compare the effect of different concentrations of both pure and nano-capsule LGO on the life table parameters of *S. frugiperda* using an age-stage and two-sex life table method. The findings of this study may contribute to producing an environmentally friendly and efficient bio-insecticide for *S. frugiperda* integrated pest management.

## Material and methods

### Materials and chemicals

The essential oil extraction unit of the National Research Centre (NRC), Egypt, provided the lemongrass essential oil (*Cymbopogon citratus* (DC.) Stapf, F: Poaceae). Tween-80, Beta-Cyclodextrin (β-CD: Purity:97%, Molecular Weight: 1134.98), gum acacia powder AR (gum Arabic) were purchased from Alpha Chemika (India), and HPLC grade solvents were purchased from Sigma-Aldrich. Castor leaves (*Ricinus communis* L.) for insect feeding were sourced from the National Research Centre’s experimental station (NRCES) in El-Nubaria, Wadi El-Natrun, El-Behera Governorate, Egypt (30° 29′ 54.22″ N 30° 19′ 10.94″ E), where the trees grow wild.

### Gas chromatography–mass spectrometry analysis

The LGO sample was dissolved in chloroform and injected into GC. The mass spectrometer detector (5977A) and gas chromatograph (7890B) were installed in the GC–MS system (Agilent Technologies) at the Central Laboratories Network, NRC, Egypt. A DB-5MS column (30 m × 0.25 mm internal diameter) and 0.25 μm film thickness were fitted to the GC. Hydrogen was used as the carrier gas during the analyses, with a splitless flow rate of 3.0 mL/min., and an injection volume of 0.2 µL. The program for temperature was configured as follows: 40 °C for one minute; 200 °C (10 °C/min) and maintained for one minute; 220 °C (20 °C/min) and maintained for one minute; 320 °C (30 °C/min) and maintained for three minutes. At 250 °C and 320 °C, the injector and detector were maintained. By utilizing a spectral range of *m/z* 50–550 and a solvent delay of three minutes, mass spectra were obtained by electron ionization (EI) at 70 eV. The mass temperature and Quad were 230 °C and 150 °C, respectively. Different components have been identified by comparing the spectrum fragmentation pattern with those recorded in the Wiley and NIST Mass Spectral Library data^21^.

### Synthesis of nano-capsules

The preparation of lemongrass essential oil encapsulated into β-cyclodextrin and Gum Arabic (LGO: β-CD/GA) was performed according to Emamjomeh et al.^22^ with modifications. Wall material consisted of β-CD and GA (50% solids) at the ratio of 1:1 β-CD: GA (w/w), whereas the LGO was utilized as a dispersed phase. Initially, β-CD and GA solid phase were dissolved in distilled water (50 g w/w) and heated at 60 °C for 45 min. To guarantee that the polymer molecules were fully saturated, the solution was then covered and left overnight at room temperature. After 24 h, the obtained solution was separated into five equal parts, and different concentrations of LGO were mixed separately with β-CD/GA solutions at the rate of 0.8, 0.4, 0.2, 0.1, and 0.0/20 g to obtain concentrations of 4.0, 2.0, 1.0, 0.5, and 0.0% (w/w). To obtain the final nano-capsules, the emulsion vessels were put in an ice-filled beaker and sonicated for 5.0 min at an amplitude of 70% (Ningbo Haishu Kesheng Ultrasonic Equipment Co., Ltd, China).

### Characterization of LGO:β-CD/GA nano-formulations

#### DLS

The Malvern Zetasizer Nano-ZS (Malvern, Worcestershire, UK) was utilized to determine the dynamic light scattering (DLS) measurements such as Z-Average, polydispersity index (PDI), and zeta potential of nano-capsules. A dilution of nano-capsule suspensions 100-fold in deionized water was prepared for each concentration. Samples were measured at a fixed angle of 173° and 25 °C following a two-minute equilibration period, and three repeat measurements were conducted.

#### Encapsulation efficiency

To estimate the encapsulation efficiency of LGO incorporated with β-CD/GA inclusion complex, 1 mL of each nano-capsule formulation was centrifuged in triplicates at 10,000 rpm using a cooling centrifuge (NEYA 16R, India) for 15 min at 4 °C. Then, 200 µL of the supernatants were collected, and the volume was completed with ethanol to 1 mL (5 × dilution) for calculation of their oil content, which corresponds to the un-entrapped oil then measured using JENWAY 6305 Spectrophotometer (Staffordshire, UK) at wavelength of 417 nm. Total oil concentration in the formulation was similarly measured using the same method but by dissolving 100 µL of formulation in 900 µL of ethanol (10× dilution)^23^. The encapsulation efficiency was calculated by subtracting the un-entrapped oil from the total oil content and calculating the difference as a percentage of the total oil. The following is the analytical curve that was used for measurements:

$${\text{y }} = \, 0.0{\text{166x }} - \, 0.0{539 }\left( {{\text{R}}^{{2}} \, = \, 0.{9998}} \right)$$

$${\text{x}} = {\text{the}}\,{\text{absorbance}}\,{\text{at}}\,{417}\,{\text{nm}},{\text{ y}} = {\text{the}}\,{\text{concentration }}\left( {\mu {\text{L}}/{\text{mL}}} \right)$$

#### Insect

The colony of *S. frugiperda* was established at the laboratory of the Pests and Plant Protection Department, NRC, Egypt. The larvae were placed individually in plastic containers (25 mL) and reared on castor leaves *Ricinus communis* L. until the pupal stage^6^. The resultant pupae were isolated daily and placed in separate jars until the emergence of moths. The newly emerged moths were transferred to glass jars (1L) for oviposition. Each jar was supplied with paper strips for laying eggs, and moths were fed on a cotton tuft moistened with honey solution (10%). Before conducting the experiments, *S. frugiperda* was maintained in stock culture over ten generations without being exposed to pesticides. Culture and experiments were kept under laboratory conditions of 26 ± 2 °C, 66 ± 5% RH, and 16L: 8D photoperiod. Male–female pairs of recently emerged moths were released into 1L glass jars that were provided with paper strips to obtain *S. frugiperda* larvae of the same age. After the eggs were laid, paper strips containing the eggs were picked up, put in 10 cm-diameter Petri dishes, and observed until the larvae hatched.

#### Life table study

Life table parameters were studied to estimate the long-term toxic effects of nano-formulated and non-formulated LGO against *S. frugiperda* using the dipping technique. In a preliminary experiment, LGO was used at a concentration of 5.0% against the 1st larval instar, which resulted in 100% mortality after 24 h. Thus, in this study, 4.0, 2.0, 1.0, and 0.5% of LGO prepared in Tween-80 (1 mL) were used. Castor oil leaf discs with a diameter of 2.5 cm were dipped in selected concentrations of pure and nano-capsules for two minutes and then allowed to air dry at room temperature. Newly hatched larvae of *S. frugiperda* 1st instar were transferred using a fine brush to glass Petri dishes and allowed to feed separately on treated leaf discs. Twenty replicates of the experiment were carried out for each concentration, and each replicate had one larva released. The control larvae were fed on leaf discs treated with water (1.0% Tween-80 solution). The larvae provided the treated food for 96 h, then were fed on untreated food. The potential toxic effect of wall material (β-CD/GA) was tested in a preliminary experiment, and results indicated no mortality in *S. frugiperda* treated larvae after 96 h of exposure.

The lifecycle of *S. frugiperda* was divided into distinct developmental stages: eggs, larvae, pupae, and adults. The development time and survival or mortality for each stage were observed daily. The survived pair (1male:1female) of newly emerged moths was released into a glass vial to count the number of eggs laid by each female as previously described. Following the recording of the daily laid eggs, paper strips were replaced. Developmental parameters, including the total number of eggs laid each day and adult longevity, including the pre-, ovi-, and post-oviposition periods, were recorded until the insects died.

### Statistical analysis

Using SPSS version 20.0, mean values of physical characterization, mortality, and developmental parameters were compared through the one-way analysis of variance (ANOVA) and Duncan’s test (*P* < 0.05). The Finney^24^ probit analysis method was used to determine the lethal concentration values (LC_50_, LC_99_). The age-stage, two-sex life table concept was used to analyze raw data on female fecundity, longevity, and developmental features^25–27^ using TWOSEX-MS Chart software^28^. Using the bootstrap method (m = 10,000), the means and standard errors of the life table parameters were calculated^29^. The following parameters were evaluated and calculated:

1. APOP or adult pre-oviposition period: The time interval from an adult female’s emergence to the start of the first oviposition.
2. TPOP or total pre-oviposition period: The period between birth and the start of oviposition.
3. S_x_,_j_ or age-stage-specific survival rates: The possibility that a newborn will live to age x and stage j.
  $$Sxj=\frac{nxj}{n01}$$
4. l_x_ or age-specific survival rate: The probability that a newborn egg will live to age x.
  $$lx={\sum }_{j=1}^{m}Sxj$$

m is the number of stages.

(5) m_x_ or the age-specific fecundity: The average number of eggs an individual produced at age x.
  $$m_{x} = \frac{{the \mathop \sum \limits_{j = 1}^{m} Sxjfxj{ }}}{{the \mathop \sum \limits_{j = 1}^{m} Sxj{ }}}$$
(6) l_x_m_x_ or age-specific maternity: The product of l_x_ and m_x_.
(7) e_xj_ or age-stage-specific life expectancy: The time that an individual of age x and stage y is anticipated to live.
  $$e_{xj} = the \mathop \sum \limits_{j = 1}^{m} \mathop \sum \limits_{j = 1}^{m} S^{\prime}ij$$

S’ij is the probability that an individual of age x and stage y will survive to age i and stage j.

(8) v_xj_ or age-stage-specific reproductive value: The contribution of individuals of age x and stage y to the future population.
  $$v_{xj} = \frac{{e^{{ - r\left( {x + 1} \right)}} }}{Sxj}\mathop \sum \limits_{j = 1}^{m} e^{{ - r\left( {x + 1} \right)}} \mathop \sum \limits_{j = y}^{m} S^{\prime}ijfij$$
(9) r or intrinsic rate of increase.
  $${\sum }_{x=0}^{\infty }{e}^{-r(x+1)}{l}_{x}{m}_{x}=1$$
(10) λ or the finite rate of increase.
  $$\uplambda ={\text{e}}^{r}$$
(11) R_0_ or the net reproductive rate: The average number of offspring that an individual can produce during their lifetime.
  $${R}_{0}={\sum }_{x=0}^{\infty }{l}_{x}{m}_{x}$$
(12) T or the mean generation time: The time it takes for a population to grow to R_0_-fold of the number at the stable age-stage distribution.
  $$T=\frac{\text{ln} {R}_{0}}{r}$$
(13) DT or doubling time: The time it takes for a population to double in size, or the number of days required by a population to double.
  $$\text{DT}=\text{ln}2/rm$$

## Results

### Phytochemical screening of lemongrass essential oil

**Fig. 1:**
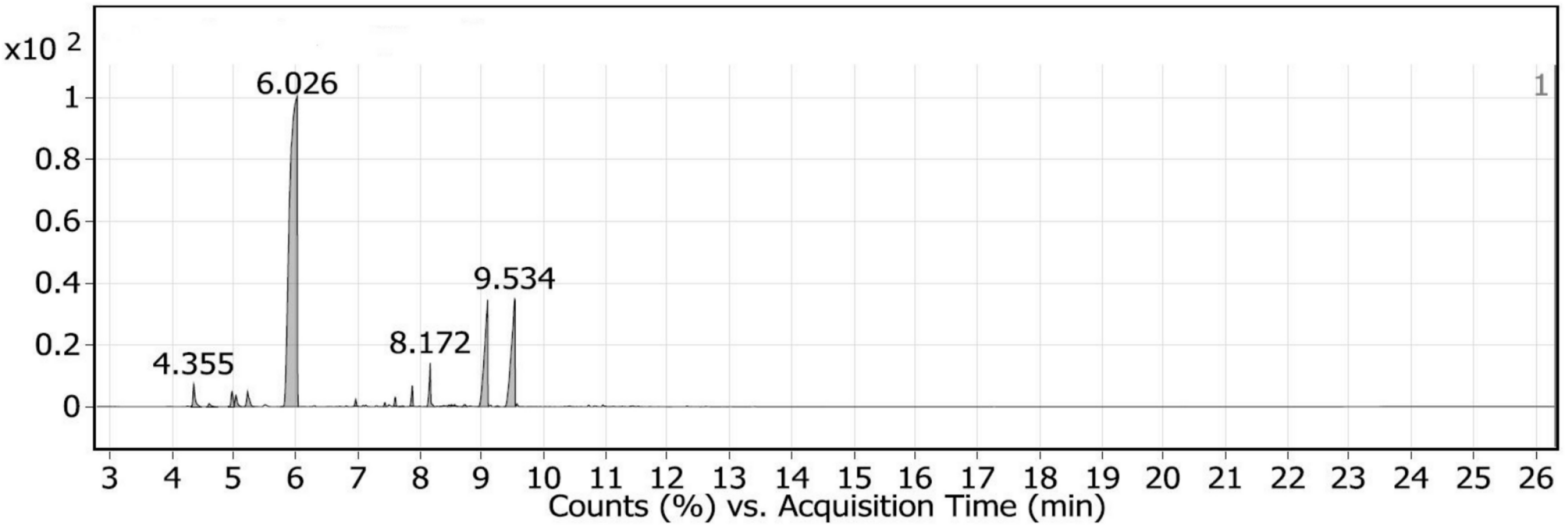
GC–MS chromatogram of lemongrass essential oil.

The GC–MS spectra of LGO (Fig. 1) indicated that among twenty identified compounds, D-Limonene was the most abundant volatile (69.55%), followed by E-Citral (11.85%) and Z-Citral (10.26%). Isogeranial, alpha-Pinene, 5-Hepten-1-yne, 6-methyl, and Sabinene were found at 1.9, 1.4, 1.11, and 0.9%, respectively. Additional compounds such as Isoneral, beta-pinene, Linalool, and Camphene were also detected as minor constituents (Table 1).

**Table 1:** Main phytoconstituents of lemongrass essential oil.

Peak	RT	Compound	Area sum %
1	4.355	alpha.-Pinene	1.4
2	4.607	Camphene	0.21
3	4.973	Sabinene	0.9
4	5.036	beta.-Pinene	0.71
5	5.225	5-Hepten-1-yne, 6-methyl	1.11
6	6.026	D-Limonene	69.55
7	6.97	Linalool	0.3
8	7.439	CIS-LIMONENE OXIDE	0.13
9	7.605	6-Octenal, 7-methyl-3-methylene-	0.35
10	7.725	Citronellal	0.03
11	7.88	Isoneral	0.79
12	8.172	Isogeranial	1.9
13	8.389	.alpha.-Terpineol	0.06
14	8.475	(−)-trans-Isopiperitenol	0.09
15	8.515	Decanal	0.09
16	8.567	Carveol	0.11
17	8.727	(−)-cis-Isopiperitenol	0.13
18	9.099	Z-Citral	10.26
19	9.253	2-Cyclohexen-1-one, 3-methyl-6-(1-methylethyl)-	0.03
20	9.534	TRANS GERANIAL/E-Citral	11.85

### Physical characterization of LGO:β-CD/GA nano-capsules

Data in Table 2 reveals that the concentration of LGO affected the DLS measurements. Higher concentrations produced larger nano-capsules, whereas the lowest concentration (0.5%) produced the smallest particle size of 279.63 nm. The values of PDI and zeta potential for tested formulations ranged from 0.36 to 0.58, and from − 17.46 to − 11.08 mV, respectively. The encapsulation efficiency was also affected by the oil concentration; the highest percentage (83.59 ± 0.34%) was obtained by using 0.5% oil concentration, whereas 4.0% oil concentration resulted in 51.94 ± 0.8% encapsulation efficiency.

**Table 2:** Physical properties and encapsulation efficiency of LGO:β-CD/GA at different oil concentrations.

Nano-capsules with oil conc.%	Particle size (nm)	PDI	Zeta potential (mV)	Encapsulation efficiency%
4.0	471.93 ± 20.73a	0.45 ± 0.01ab	− 13.60 ± 1.41ab	51.94 ± 0.84c
2.0	444.53 ± 39.97a	0.46 ± 0.03ab	− 11.08 ± 1.62a	55.91 ± 1.08b
1.0	416.16 ± 56.07a	0.58 ± 0.06a	− 13.46 ± 1.80ab	57.20 ± 0.22b
0.5	279.63 ± 36.64b	0.36 ± 0.02b	− 17.46 ± 1.58b	83.59 ± 0.34a
F	4.47*	4.52*	2.68^ns^	407.19**

Mean (± SE) values with similar letters within the same column are not significantly different (*P* < 0.05) (ANOVA) (Duncan test). * Significant, ^ns^Not significant, ^**^ Highly significant.

### Bioassay

#### Toxicity to *Spodoptera frugiperda*

**Fig. 2:**
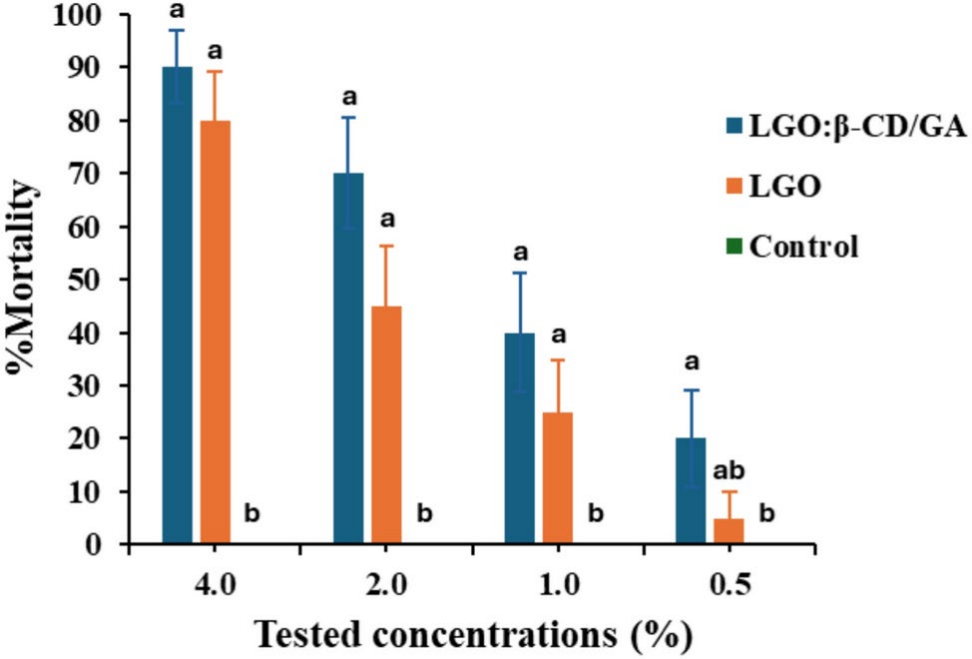
Larval mortality of *Spodoptera frugiperda* treated with different concentrations of lemongrass essential oil pure (LGO) and nano-capsules (LGO:β-CD/GA) for 96 h. Mean (± SE) values with similar letters within the same concentration are not significantly different (P < 0.05) (ANOVA) (Duncan test).

The data displayed in Fig. 2 demonstrated that when the concentration of pure oil and nano-capsules increases, it increases the mortality rate of *S. frugiperda-*treated larvae. Compared to pure LGO, nano-capsules produced higher but not statistically significant percentages of larval mortality when tested at four concentrations. Exposure to 4.0% of LGO:β-CD/GA and LGO resulted in mortality rates of 90 ± 6.88 and 80 ± 9.18%, respectively, compared to untreated larvae (F: 55.48; *P* < 0.0001). Following treatment with 2.0% of LGO:β-CD/GA and LGO led to a 70 ± 10.51 and 45 ± 11.41% mortality percentage, respectively (F:15.67; *P* < 0.0001). Treatment with 1.0 and 0.5% concentrations of LGO:β-CD/GA resulted in 40 ± 11.23 and 20 ± 9.17% mortality percentages, respectively, whereas at the same concentrations of LGO produced mortality rates ≤ 25% (F: 5.44, *P* < 0.0001 and F: 2.97, *P* < 0.05, respectively). Results in Table 3 demonstrated that after 96 h, the LC_50_ and LC_99_ values obtained from treating *S. frugiperda* with LGO:β-CD/GA were lower than those obtained after LGO, suggesting that the nano-capsules were more toxic.

**Table 3:** Toxicity of lemongrass essential oil pure (LGO) and nano-capsules (LGO:β-CD/GA) against *Spodoptera frugiperda* treated with different concentrations for 96 h.

	LGO pure	LGO:β-CD/GA
LC_50_ (95% Confidence Limit (LL-UL)	2.48 (1.98–3.17)	1.52 (0.94–2.07)
LC_99_ (95% Confidence Limit (LL-UL)	6.16 (4.90–8.97)	5.38 (4.14–8.52)
Slope (± SE)	0.63 ± 0.12	0.60 ± 0.13
X^2^	1.29	1.49

LC: (%); LL-UL: lower limit–upper limit.

#### Developmental time

**Fig. 3:**
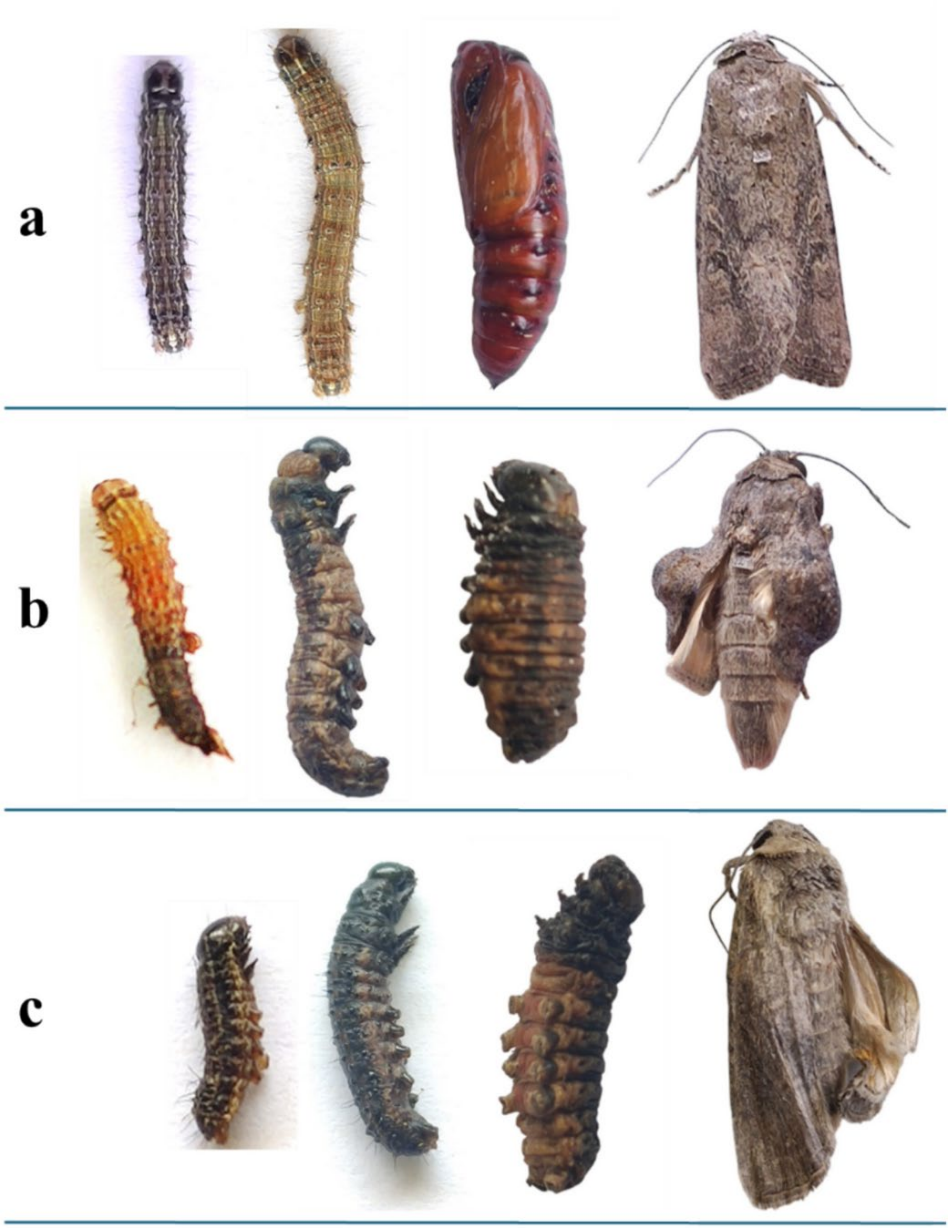
Showing untreated *Spodoptera frugiperda* larvae, pupa and adult (**a**), and malformed larvae (failed to ecdyse), larval-pupal intermediate, and malformed adult resulted from 1.0% treatment with nano-capsules (LGO:β-CD/GA) (**b**) and pure lemongrass oil (**c**)

The results demonstrated that the development time of *S. frugiperda* was significantly impacted by the toxicity of both pure LGO and nano-capsules treatments. In both formulations, as oil concentration increases it also increases the toxic effect (Table 4). However, the longest larval and pupal durations were produced by treatment with LGO:β-CD/GA. The larval duration was significantly prolonged by all tested concentrations of LGO:β-CD/GA, and this was followed by 4.0 and 2.0% of LGO. While there was no significant difference between the control and LGO at 1.0 and 0.5%. The four tested concentrations of LGO:β-CD/GA also significantly extended the pupal duration of *S. frugiperda*, while LGO treatment did not show a significant increase in pupal duration compared to the control. The shortest adult longevity was 1.50 ± 0.50 days produced by exposure to 4.0% LGO:β-CD/GA compared to 6.50 ± 0.50 and 7.50 ± 0.28 days recorded for 4.0% LGO and control, respectively. Since only one female and male individual survived in 4.0% LGO:β-CD/GA treatment, their longevity values were excluded from the statistical analysis of female and male longevity. Accordingly, the longest total longevity, 48.0 ± 0.0 and 47.83 ± 1.01 days, were obtained by 4.0 and 2.0% LGO:β-CD/GA, respectively, followed by 1.0 and 0.5% concentration. There was no significant difference in total longevity between 4.0 and 2.0% of LGO, as well as between 1.0 and 0.5 LGO and control. It is noticeable that the application of both LGO and LGO:β-CD/GA treatments disrupted the normal growth of *S. frugiperda*, leading to incomplete moults in which the larvae were unable to ecdyse. Additionally, larval-pupal intermediate and malformed adults were also observed (Fig. 3).

**Table 4:** Effect of lemongrass oil pure (LGO) and nano-capsules (LGO:β-CD/GA) on the development of *Spodoptera frugiperda.*

Tested formulation (oil conc.%)		Duration (days)							
		Egg	Larva	Pupa	Adult	Pre-adult	Total longevity	Female longevity	Male longevity
LGO	4.0	2.65 ± 0.10ab	17.75 ± 0.47e	7.50 ± 0.29cd	6.50 ± 0.50b	27.75 ± 0.75e	34.25 ± 0.75c	7.00 ± 1.00a	6.00 ± 0.00bc
	2.0	2.75 ± 0.09ab	15.00 ± 0.76f.	7.72 ± 0.23cd	7.81 ± 0.26a	25.36 ± 0.95f.	33.18 ± 0.94c	7.80 ± 0.20a	7.83 ± 0.48a
	1.0	2.45 ± 0.11ab	12.75 ± 0.32g	6.81 ± 0.16d	7.50 ± 0.15a	22.00 ± 0.27g	29.50 ± 0.25d	7.85 ± 0.14a	7.23 ± 0.22ab
	0.5	2.55 ± 0.11ab	11.78 ± 0.24g	7.10 ± 0.18d	7.94 ± 0.16a	21.47 ± 0.29g	29.42 ± 0.38d	7.75 ± 0.16a	8.09 ± 0.25a
LGO:β-CD/GA	4.0	2.65 ± 0.10ab	33.50 ± 0.50a	10.00 ± 0.00a	1.50 ± 0.50d	46.50 ± 0.50a	48.00 ± 0.00a	1.00^	2.00^
	2.0	2.75 ± 0.09ab	31.50 ± 0.67b	9.34 ± 0.21ab	4.34 ± 0.42c	43.50 ± 0.67b	47.83 ± 1.01a	3.66 ± 0.33b	5.00 ± 0.57cd
	1.0	2.55 ± 0.11ab	28.41 ± 0.59c	9.25 ± 0.25ab	4.34 ± 0.14c	40.41 ± 0.62c	44.75 ± 0.62b	4.33 ± 0.21b	4.34 ± 0.21d
	0.5	2.55 ± 0.11ab	24.56 ± 0.60d	8.37 ± 0.25bc	7.81 ± 0.18a	35.50 ± 0.65d	43.31 ± 0.74b	7.71 ± 0.18a	7.88 ± 0.30a
Control	0.0	2.90 ± 0.06a	12.15 ± 0.31g	8.15 ± 0.23c	7.50 ± 0.28a	23.20 ± 0.42g	30.70 ± 0.50d	7.44 ± 0.24a	7.54 ± 0.49a
F		1.70^ns^	222.93**	13.51**	38.49**	210.47**	126.66**	40.96**	10.75**

Mean (± SE) values with similar letters within the same column are not significantly different (P < 0.05) (ANOVA) (Duncan test).

**Highly significant, ^ns^Not significant, ^ values of 1.0 and 2.0 refer to the longevity of one individual female and one individual male.

#### Reproduction

The toxic effects of lemongrass oil as pure or nano-capsule formulations on the APOP, TPOP, and fecundity of *S. frugiperda* are presented in Table 5. Nano-capsules of LGO:β-CD/GA significantly affected the reproduction of *S. frugiperda*. The APOP, TPOP, and fecundity for 4.0% LGO:β-CD/GA were zero. The APOP did not differ significantly between *S. frugiperda* treated with four concentrations of LGO and 0.5% LGO:β-CD/GA in comparison to control. However, 2.0 and 1.0% LGO:β-CD/GA reduced significantly APOP. A similar finding was observed for TPOP, which was shorter for 2.0 and 1.0% LGO:β-CD/GA. A significant reduction in fecundity of *S. frugiperda* was obtained after treatment with 2.0 and 1.0% LGO:β-CD/GA, followed by 4.0, 2.0, and 1.0% LGO. Following treatment with 0.5 and 1.0% LGO, fecundity was 143.75 ± 15.70 and 110.85 ± 7.95 eggs/female, respectively, compared to 211.66 ± 16.12 eggs/female for the control group.

**Table 5:** Effect of lemongrass oil pure (LGO) and nano-capsules (LGO:β-CD/GA) on APOP, TPOP, and fecundity of *Spodoptera frugiperda.*

Tested formulation (oil conc.%)		APOP	TPOP	Fecundity
LGO	4.0	2.00 ± 0.00a	29.00 ± 0.00ab	32.50 ± 7.50de
	2.0	1.60 ± 0.24a	26.80 ± 1.39ab	63.00 ± 16.87d
	1.0	1.85 ± 0.14a	23.71 ± 0.47ab	110.85 ± 7.95bc
	0.5	1.62 ± 0.18a	23.50 ± 0.32ab	143.75 ± 15.70b
LGO:β-CD/GA	2.0	0.66 ± 0.66b	15.33 ± 15.33b	5.00 ± 5.00e
	1.0	0.66 ± 0.33b	21.66 ± 9.69b	7.50 ± 3.59e
	0.5	2.14 ± 0.26a	38.14 ± 0.67a	69.28 ± 6.26cd
Control	0.0	1.66 ± 0.16a	24.22 ± 0.46ab	211.66 ± 16.12a
F		4.03**	2.08**	29.58**

Mean (± SE) values with similar letters within the same column are not significantly different (*P* < 0.05) (ANOVA) (Duncan test). **Highly significant.

#### Population parameters

The effects of LGO and LGO:β-CD/GA on life table parameters of *S. frugiperda* are given in Table 6. Only one female survived 4.0% LGO:β-CD/GA treatment, consequently, the intrinsic rate of increase (r), net reproductive rate (R_0_), and gross reproductive rate (GRR) had zero value. Moreover, the population will rarely double in size if r < 0, meaning that the doubling time will be negative. The population will not increase, and there will not be a doubling time if r = 0. Significant high values of the intrinsic rate of increase, finite rate of increase, net reproductive rate, and gross reproductive rate were observed in the control group, followed by 0.5, 1.0, 2.0, and 4.0% LGO. Compared with control (0.16 ± 0.03), the intrinsic rate of increase showed significant reduction after exposure to LGO:β-CD/GA at 4.0, 2.0, 1.0, and 0.5% concentrations (0.00, 0.001 ± 0.02, 0.01 ± 0.04, and 0.07 ± 0.02 day^−1^, respectively). Furthermore, no significant variation was observed in the finite rate of increase between 0.5 and 1.0% LGO and the control group (1.18 ± 0.03 day^−1^). On the other hand, LGO:β-CD/GA showed a noticeably lower finite rate of increase (1.0, 1.00 ± 0.02, 1.01 ± 0.04, and 1.08 ± 0.03 day^−1^) in *S. frugiperda* treated with concentrations of 4.0, 2.0, 1.0, and 0.5%, respectively. *Spodoptera frugiperda* treated with 2.0, 1.0, and 0.5% LGO:β-CD/GA had much lower net reproductive rate values (1.16 ± 0.001, 2.34 ± 0.003, and 24.25 ± 0.02, respectively) than those on LGO treatment at 4.0, 2.0, 1.0, and 0.5% (3.66 ± 0.006, 15.79 ± 0.02, 38.83 ± 0.03, and 57.49 ± 0.05, respectively) and control (95.35 ± 0.07). The gross reproductive rate showed similar trends. Furthermore, compared to LGO treatment, LGO:β-CD/GA had a greater impact on the mean generation time (T), with values ranging from 39.79 to 47 days at the four tested concentrations. Conversely, 1.0% and 0.5 LGO produced mean generation times that were very close to that of the control group (26.61 ± 1.47 days).

**Table 6:** Effect of lemongrass oil pure (LGO) and nano-capsules (LGO:β-CD/GA) on population parameters of *Spodoptera frugiperda.*

Tested formulation (oil conc.%)		Intrinsic rate of increase (r/day)	Finite rate of increase (λ/day)	Net reproductive rate (R0)	Gross reproductive rate (GRR) (offspring/individual)	Mean generation time (T/day)	Doubling time (D)T
LGO	4.0	0.03 ± 0.05bc	1.03 ± 0.06bc	3.66 ± 0.006de	16.24 ± 0.01cd	30.13 ± 0.29c	29.82 ± 0.08a
	2.0	0.09 ± 0.06b	1.09 ± 0.07b	15.79 ± 0.02d	35.80 ± 0.04c	28.94 ± 4.45cd	7.40 ± 0.03b
	1.0	0.13 ± 0.04ab	1.14 ± 0.04a	38.83 ± 0.03c	50.75 ± 0.04b	26.03 ± 1.61de	5.06 ± 0.001bc
	0.5	0.15 ± 0.04a	1.16 ± 0.04a	57.49 ± 0.05b	66.39 ± 0.05b	26.17 ± 1.20de	4.56 ± 0.001c
LGO:β-CD/GA	4.0	0.00	1.00	0.00	0.00	47.00	–
	2.0	0.001 ± 0.02d	1.00 ± 0.02 cd	1.16 ± 0.001e	5.60 ± 0.008d	46.99 ± 0.00001a	–
	1.0	0.01 ± 0.04cd	1.01 ± 0.04c	2.34 ± 0.003de	6.24 ± 0.008d	44.70 ± 2.11b	–
	0.5	0.07 ± 0.02b	1.08 ± 0.03b	24.25 ± 0.02 cd	37.23 ± 0.03c	39.79 ± 2.21bc	8.95 ± 0.004b
Control	0.0	0.16 ± 0.03a	1.18 ± 0.03a	95.35 ± 0.07a	105.04 ± 0.08a	26.61 ± 1.47d	4.09 ± 0.0008c

Mean (± SE) followed by similar letters within the same column are not significantly different (*P* < 0.05), using Bootstrap method. SE values for r, λ, T (E-3).

#### Survival and fecundity

**Fig. 4.**
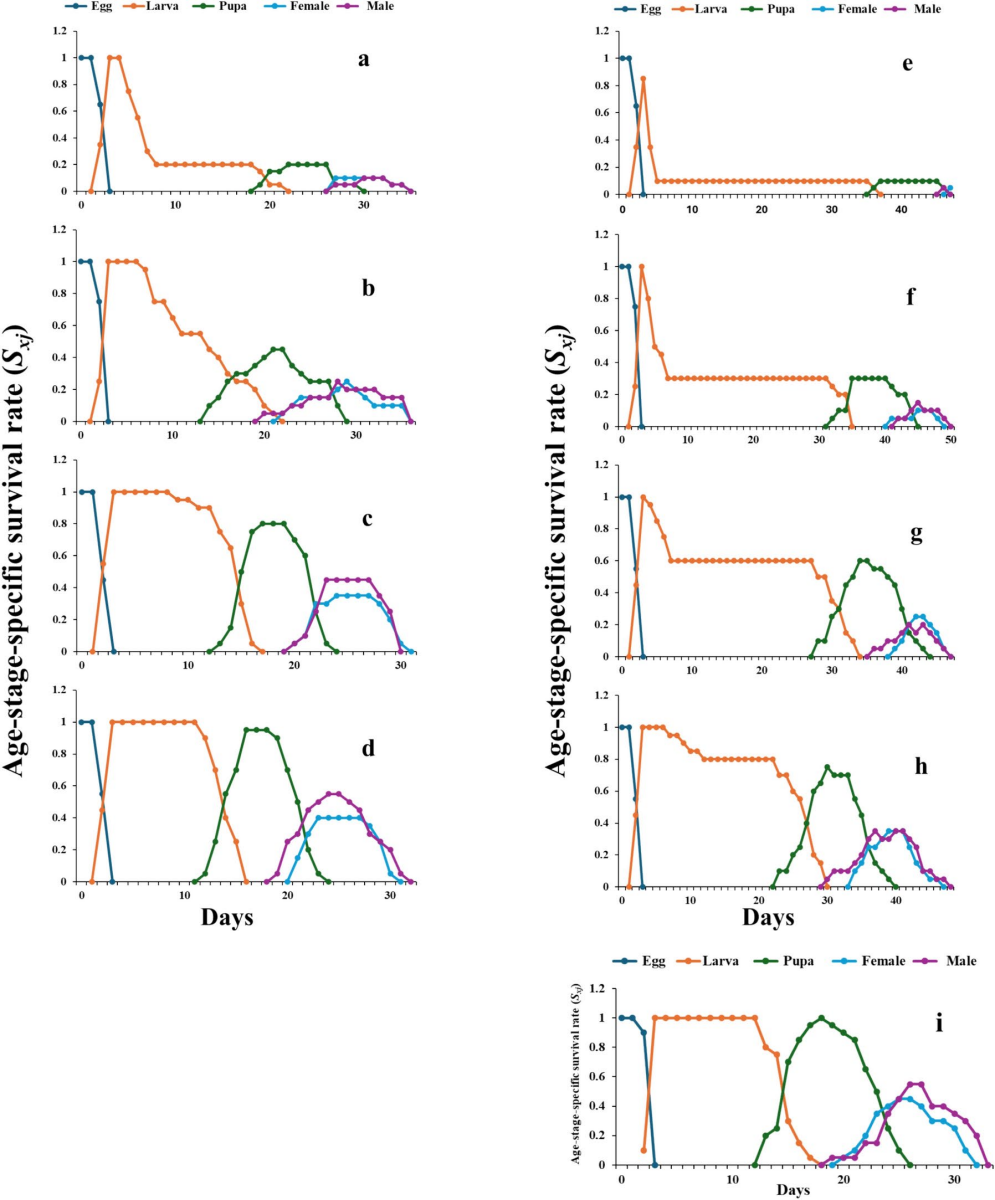
Age-stage specific survival rate of *Spodoptera frugiperda* exposed to lemongrass oil LGO [4.0% (**a**), 2.0% (**b**), 1.0% (**c**), 0.5% (**d**)] and nano-capsules LGO:β-CD/GA [4.0% (**e**), 2.0% (**f**), 1.0% (**g**), 0.5% (**h**)], compared to control (**i**).

Curves of the age-stage specific survival rate (S_xj_) in *S. frugiperda* treated with pure and nano-capsule lemongrass oil are displayed in Fig. 4. According to the data, the expectation that a newly laid egg will survive to age x tended to decrease when the parents were exposed to both LGO and LGO:β-CD/GA, with the nano-capsules causing a more pronounced decline in this probability. These curves appeared to exhibit an overlap condition because individuals developed at different rates. In particular, at 4.0 and 2.0% LGO:β-CD/GA (> 40 days), all treatment stages took longer to develop overall than the control group (33 days).

**Fig. 5.**
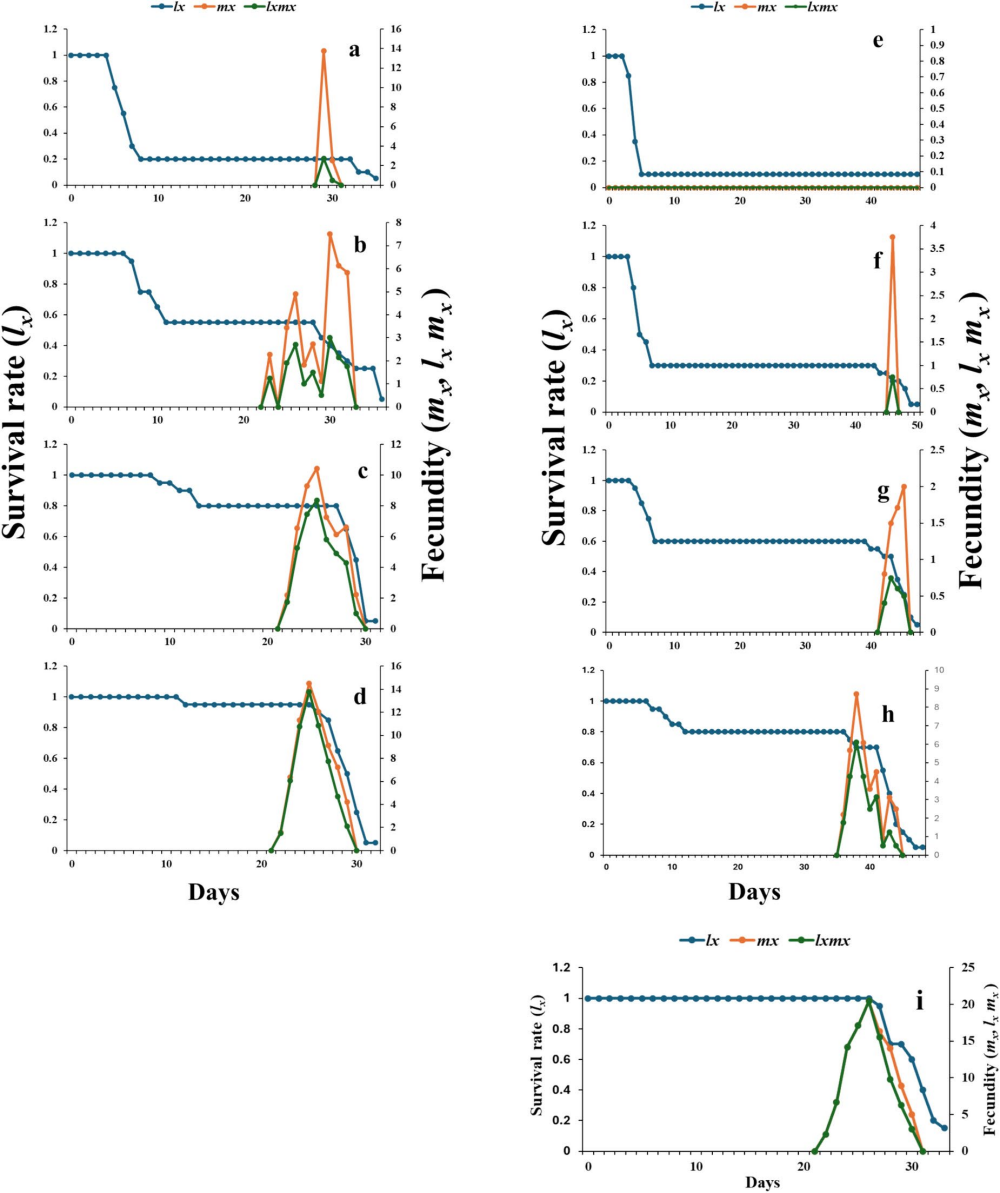
Population survival rate and fecundity of *Spodoptera frugiperda* exposed to lemongrass oil LGO [4.0% (**a**), 2.0% (**b**), 1.0% (**c**), 0.5% (**d**)] and nano-capsules LGO:β-CD/GA [4.0% (**e**), 2.0% (**f**), 1.0% (**g**), 0.5% (**h**)], compared to control (**i**).

Figure 5 displays age-specific survival rate (l_x_), fecundity (m_x_), and net maternity (l_x_m_x_) for parental generation of treated and control *S. frugiperda*. The exposure of *S. frugiperda* from the parental generation to both formulations pure and nano-capsules at different concentrations produced, in comparison to the control, a shorter period of oviposition, as well as a drop in age-specific fecundities. However, the effect of LGO:β-CD/GA, particularly at higher concentrations (4.0 and 2.0%), was significant. The 4.0 and 2.0% of LGO:β-CD/GA showed a steep decline in survivorship that began on the third and fourth day, respectively. On the fifth and ninth days, respectively, the decline was noted at the same concentrations of LGO. Conversely, no noticeable drop occurred at lower LGO concentrations. According to the m_x_ curve, reproduction started at age 29 and 23 days in 4.0 and 2.0% LGO treatments and at 22 days in 1.0 and 0.5% LGO compared to control (on day 22). The LGO:β-CD/GA treatment appeared to have a significant impact on fecundity, especially at 4.0% when m_x_ was zero. Additionally, the reproduction started on days 46, 42, and 36 for 2.0, 1.0, and 0.5%, respectively. Since l_x_ and m_x_ are the main factors that determine the l_x_m_x_ value, the l_x_m_x_ value for 4.0% LGO:β-CD/GA treatment was zero. The maximum l_x_m_x_ values for 4.0, 2.0, 1.0, and 0.5% LGO were 2.75, 3.0, 8.35, and 13.8, respectively. Compared to the control (20.45), the l_x_m_x_ values of 0.75, 0.75, and 6.10 were observed for LGO:β-CD/GA at 2.0, 1.0, and 0.5%, respectively.

#### Life expectancy and reproductive value

**Fig. 6.**
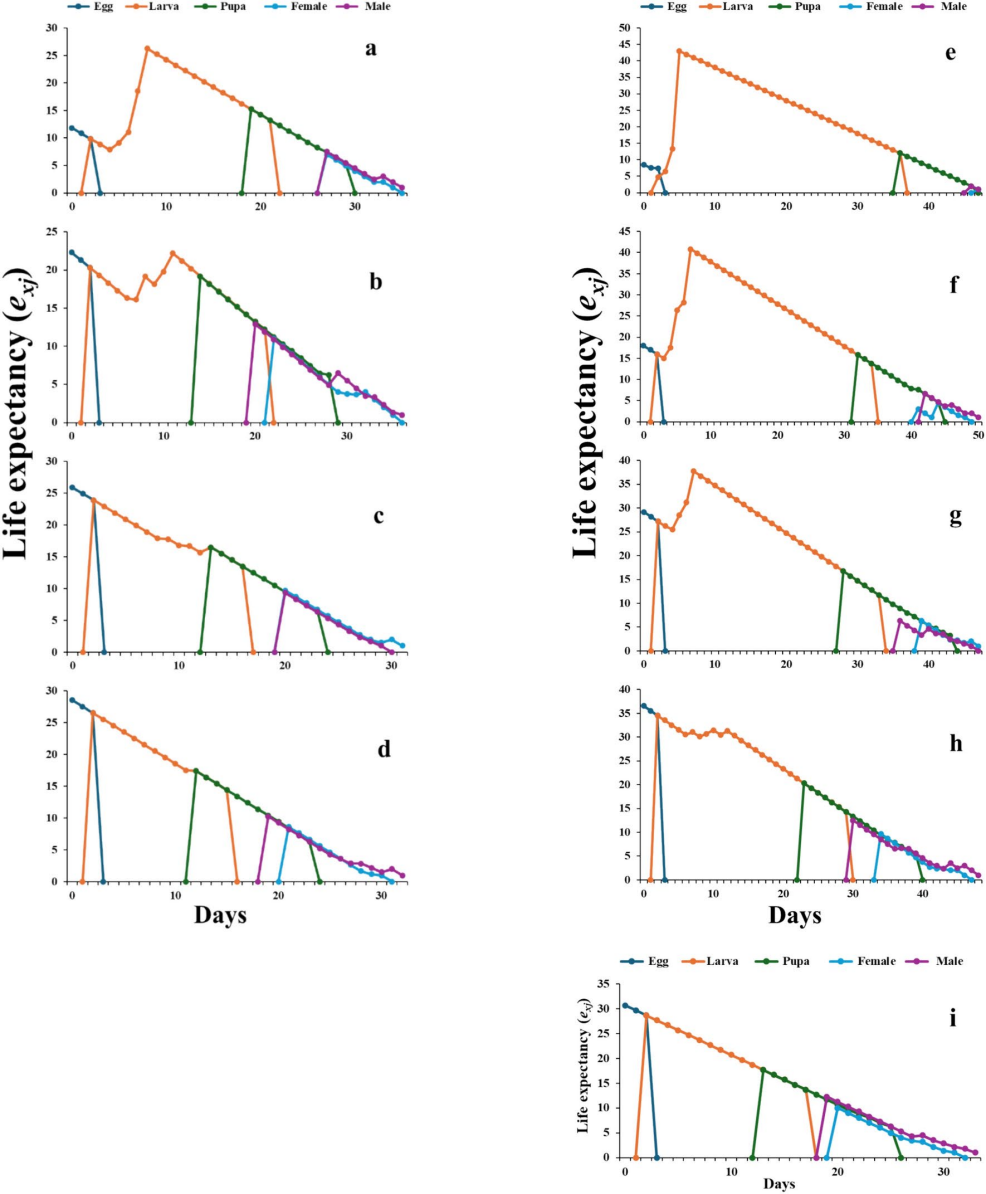
Life expectancy of *Spodoptera frugiperda* exposed to lemongrass oil LGO [4.0% (**a**), 2.0% (**b**), 1.0% (**c**), 0.5% (**d**)] and nano-capsules LGO:β-CD/GA [4.0% (**e**), 2.0% (**f**), 1.0% (**g**), 0.5% (**h**)], compared to control (**i**).

**Fig. 7.**
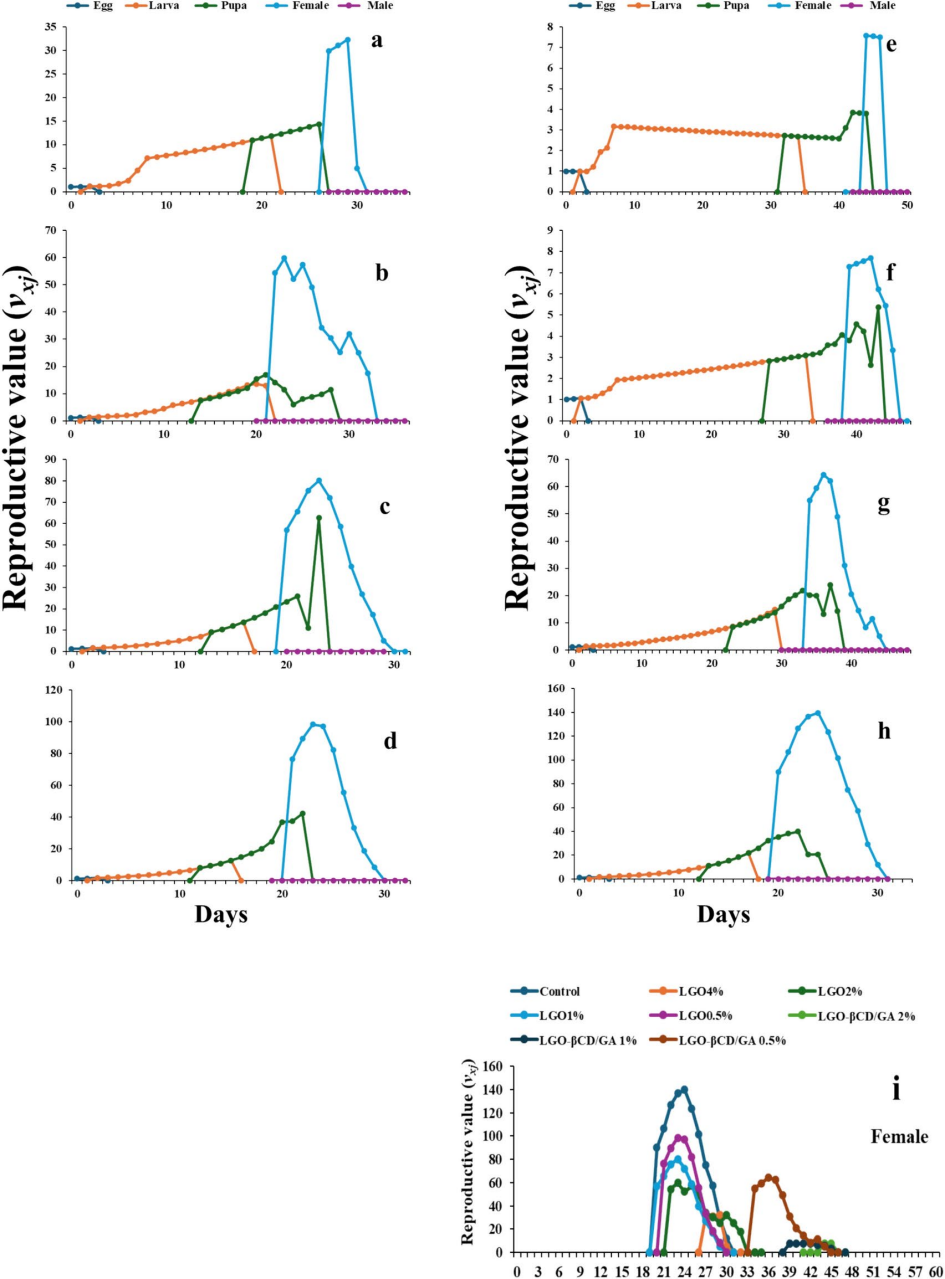
Reproductive value of *Spodoptera frugiperda* exposed to lemongrass oil LGO [4.0% (**a**), 2.0% (**b**), 1.0% (**c**), 0.5% (**d**)] and nano-capsules LGO:β-CD/GA [2.0% (**e**), 1.0% (**f**), 0.5% (**g**)], compared to control 0.5% (**h**). The reproductive value of *S. frugiperda* females exposed to all treatments is shown in (**i**).

The life expectancy (e_xj_) in all stages varied according to the treatment and compared to the control (Fig. 6). The life expectancy in the larval stage of control seemed to reduce compared with 4.0 and 2.0% LGO and LGO:β-CD/GA four tested concentrations. Additionally, LGO:β-CD/GA at 4.0% severely affected the e_xj_ in the adult stage. According to Fig. 7, all treatments influenced the age-stage-specific reproductive values (v_xj_); however, the 4.0% LGO:β-CD/GA treatment produced a v_xj_ value of zero. Additionally, it is obvious that the LGO and LGO:β-CD/GA treatments caused the (v_xj_) of the female *S. frugiperda* to tend to decrease; nevertheless, the nano-capsules showed the most notable decreases at 2.0, 1.0, and 0.5%.

## Discussion

The use of natural products like essential oils is becoming a growing trend in the agricultural sector as an alternative to chemical insecticides. Several essential oils demonstrated high toxicity to different insect species^5,30^. However, in addition to their low solubility, preparations of EO-based insecticides are usually highly degradable and unstable under harsh climatic conditions. Due to these poor physicochemical characteristics, the use of EOs as insecticides on a large scale is restricted. This work aimed to develop a nano-formulation of LGO that retains its active components while intensifying its detrimental impact on *S. frugiperda* in comparison to non-formulated oil. The GC–MS analysis identified twenty compounds in LGO. D-Limonene, E-Citral, and Z-Citral were detected as main compounds, additionally isogeranial, alpha-pinene, 5-Hepten-1-yne, 6-methyl, sabinene, isoneral, beta-pinene, linalool, and camphene compounds were also identified. Lemongrass has been found to contain α-Citral and β-Citral as main compounds^31^. Similarly, D-limonene, β-Citral, and α-Citral were the primary compounds found in lemongrass essential oil in percentages of 45.06%, 10.30%, and 9.90%, respectively^32^. This finding is consistent with earlier studies that identified the major compounds in *C. citratus* oil as citral, neral, geranyl acetate, geranial, limonene, and camphene^33,34^. However, the composition of essential oils is not homogeneous. The phytochemicals and biological activity of essential oil are complicated due to the fact that various factors, including altitude, climate, and pests, influence its composition^35^.

Bioactive substances have been incorporated using various encapsulating agents. In this work, beta-cyclodextrin (β-CD) and gum Arabic (GA) were used as wall material to nano-encapsulate LGO. One of the most widely used wall materials is β-CD, which has a hydrophilic outer surface and a hydrophobic cavity, as well as it can release its core components in a controlled release^36^. It was noted that β-CD could be effectively used to encapsulate unstable materials such as essential oils and plant-derived compounds^37^. Because of its colloid functionality, GA is a valuable agent in encapsulation procedures. The majority of starches, proteins, and carbohydrates can be combined with GA and oils to formulate more stable emulsions over a broad range of pH^38^. The stability and homogeneity of the produced nano-capsules are significantly influenced by the parameters of particle size and polydispersity index (PDI). The results showed that in a nano-capsule formulation containing 0.5% LGO, the smallest particle size of 279.63 nm was produced. Meanwhile, the mean particle size increased with the increase in oil concentration. The PDI values in this study were in the range of 0.36 to 0.58 for all tested nano-capsules. Droplets exhibit good dispersion, greater stability, and no tendency to aggregate, attributed to the low PDI values. High PDI values, on the other hand, indicate wide-range droplet dimensions^39^. In this study, zeta potential, a surface charge measurement, had negative values ranging from − 17.46 to − 11.08 mV for tested nano-capsules with oil concentrations of 4.0, 2.0, 1.0, and 0.5%. Formulations with higher (positive or negative) zeta potential values are more stable than those with lower values^40^. In this work, the smallest particle size, lowest PDI, and highest zeta potential were obtained in LGO:β-CD/GA containing 0.5% of LGO. Similar to this, Hadian et al.^41^ formed βCD: geraniol inclusion complexes with corresponding particle size, PDI, and zeta potential ranging from 111 to 258 nm, 0.16 to 0.47, and − 19 to − 21 mV, respectively. In a previous study, when *Citrus reticulata* essential oil nano-encapsulated in whey protein isolate, maltodextrin, and gum Arabic produced nano-capsules with particle size and PDI in the range of 427.35–782.09 nm and 0.26–0.47, respectively^42^. Encapsulation efficiency (%EE) was assessed to determine the amount of LGO entrapped in β-CD: GA inclusion, thereby assuring that the target insect pest receives a sufficient quantity of the bioactive substance. The properties of the wall and the core materials determine the EE%. Using 0.5% oil concentration produced the highest EE% (> 80%). However, as oil concentration increased, the EE% decreased; for the other tested concentrations (4.0, 2.0, and 1.0%), the EE% ranged from 51.94–57.20%. Similar to this finding, a larger mean diameter (309.8–402.2 nm) and a lower encapsulation efficiency (21.1–47.7%) were observed by using the increased ratio of chitosan: essential oil. The maximum encapsulation efficiency was obtained with a lower ratio (1:0.1w/w)^43^. The EE% of carvacrol and linalool loaded in β-cyclodextrin/chitosan nanoparticles was 93.9 ± 0.58% and 86.9 ± 0.9%, respectively^44^. Also, Saffron and cinnamon essential oils encapsulated in βCD/GA inclusion resulted in EE% of 72–98% and > 80%, respectively^45,46^.

The results of the present study indicate that LGO, both in non-formulated and nano-formulated preparations, exhibited significant toxicity against *S. frugiperda*. As the concentration of oil increased, it also raised the mortality rate in the treated larvae. The insecticidal activity of LGO could be due to the presence of several components known to have biological activities. These major components of LGO have been reported to have high insecticidal activity; for instance, LGO, citral, and geranyl acetate (LD_50_ = 5.17, 4.17, and 7.21 μg insect^–1^, respectively) were reported to exert lethal and sublethal effects on stored grain pest, *Ulomoides dermestoides*^34^. Limonene also is another monoterpene that is frequently present in the composition of many essential oils. Using AzamaxVR and its main constituents, such as limonene, demonstrated a highly toxic effect against *S. frugiperda* larvae^47^. The insecticidal action of many essential oils usually exerts their toxic effects through synergism between the contained phytocompounds, which improves their effectiveness^48^. In the same way, it was found that when essential oils of cinnamon, lemongrass, and rosemary were tested against *S. frugiperda*, the lemongrass EO had the highest rate of pest mortality^6^. However, compared to pure LGO, the nano-formulated oil performed better and had higher mortality rates and lower LC_50_ and LC_99_ values in *S. frugiperda* larvae through enhanced stability and bioavailability. In addition to facilitating controlled and continuous release for long-term protection, the encapsulation technique appears to shield essential oils from interactions with surrounding oxygen, evaporation, and other changes that could impair their biological activities^49^. As far as we know, few research works have examined the impact of encapsulated essential oils on *S. frugiperda*. It was found that *Azadirachta indica* extracts loaded into microcapsules made of sugarcane bagasse lignin were effective against *S. frugiperda* and *Diatraea saccharalis*, killing 100% of the insects in less time than the controls^50^. Also, *Agrotis ipsilon* and *Phthorimaea operculella* were more severely affected by nano-formulations of essential oils of lemon peel, cinnamon, and lemongrass than by pure oils^46,51,52^.

Plant products delay the development of insects because of their toxic effects. In the current study, exposure of *S. frugiperda* to LGO:β-CD/GA significantly prolonged the larval and pupal duration. Also, the shortest adult longevity was produced by treatment with 4.0% LGO:β-CD/GA. The two main factors contributing to the expanding number of insects are fecundity and adult fitness^53^. The longest total longevity, according to our results, was achieved by 4.0 (48.0 days) and 2.0% (47.83 days) LGO:β-CD/GA. Also, the reproduction of *S. frugiperda* was severely affected by LGO:β-CD/GA treatment, particularly 4.0% concentration, which resulted in zero for APOP, TPOP, and fecundity. While the maximum number of eggs was recorded after treatment with 0.5 (143.75 ± 15.70) and 1.0% (110.85 ± 7.95 eggs/female) of pure LGO. In previous studies, the treated *S. frugiperda* exhibited a notable reduction in adult longevity, fecundity, and fertility as well as an increase in larval duration upon exposure to *Foeniculum vulgare*, long pepper, and clove oil^54^. With a viability rate of over 90%, the number of eggs deposited by untreated *S. frugiperda* females was noticeably greater than that of adults treated with citronella oil. None of the eggs laid by the treated adults hatched, making them non-viable^55^. Essential oils from *Lippia origanoides*, *C. winterianus*, and *C. citratus* showed insecticidal properties on the eggs, 3^rd^ larval instars, and pupae of *S. frugiperda*^56^.

It is obvious that opposed to pure LGO, nano-capsules treatment resulted in an unsuccessful development of *S. frugiperda* since it prolongs the life cycle of the insect while reducing fecundity. Several studies have reported that the release of essential oils nano-formulations is slower than pure essential oils, which may account for the effectiveness of nano-capsules^57^. It has been shown that encapsulating lemongrass essential oil with maltodextrin may maintain the concentration of the main components without drastically affecting the oil’s quality. The volatile composition of the encapsulated essential oil was mostly retained after encapsulation, with a greater citral content^58^. Furthermore, Phunpee et al.^37^ examined the encapsulation of LGO using a variety of cyclodextrins, including β-cyclodextrin and observed that the encapsulation procedure had no discernible effect on the oil’s chemical constitution. These results imply that LGO can be efficiently encapsulated by employing gum Arabic and β-cyclodextrin without undergoing appreciable alterations to its chemical profile. Compared to pure oil, the nano-formulation of cinnamon essential oil resulted in noticeably longer periods in the larval and pupal stages of potato tuber moth^42^. Nano-encapsulated curcumin and carvacrol exhibited significantly higher mortality rates on *S. frugiperda*, *S. cosmioides*, *S. eridania*, and *H. armigera*^59^. When applied to *S. frugiperda*, nano-encapsulated azadirachtin demonstrated a more rapid and potent effect than bulk azadirachtin treatment^60^.

The parameters of the life table are influenced not only by the species of hosts but also by the pesticides and environmental factors^61^. The reproductive traits r, λ, R_0_, and GRR hold significance in explaining how food affects the fitness of insect pests^62^. Different kinds of nourishment were found to have an impact on the insect’s ability to reproduce; accordingly, insects with poor nutrition may grow smaller, produce fewer eggs, and be less able to reproduce^63^. One essential demographic measure for estimating the growth of an animal’s population is the intrinsic rate of increase (r); a greater value corresponds to faster development^64^. There is insufficient data on how the essential oils affect the life features of *S. frugiperda* when two sex tools are used. However, in the current study, the population growth of *S. frugiperda* decreased as a result of exposure to nano-capsules at varying concentrations. Nano-capsules appeared to lower all the parameters of the insect life table, such as net reproductive rate, intrinsic rate of increase, and finite rate of increase. These findings indicate that LGO:β-CD/GA treatments inhibited the population growth of *S. frugiperda*. It was reported that sublethal concentrations (LC_25_) of pure and nano-capsulated oil of *Thymus daenensis* exhibited significant effects on the life table features of *Brevicoryne brassicae* when tested under laboratory conditions^65^. According to their results, the intrinsic rate of increase, the finite rate of increase, and the net reproductive rate were significantly lower (0.16 day^−1^, 1.17 day^−1^, and 5.24 nymphs) for oil nano-capsules than aphids in pure formulation treatment (0.21 day^−1^, 1.24 day^−1^, and 9.84 nymphs). The *S. frugiperda* individuals’ varying developmental rates in stage differentiation were indicated by the overlap in the s_xj_ curves. The life expectancy and reproductive value in individuals of the same age range but at different stages are demonstrated by the e_xj_ and v_xj_ curves. Without assuming that the population attains a constant distribution of age-stage, the e_xj_ is computed using the s_xj_. As a result, it can be used to anticipate a population’s probability of survival under those circumstances. The life expectancy (e_xj_) and age-specific reproductive values (v_xj_) of *S. frugiperda* were influenced by all treatments in this study. However, LGO:β-CD/GA especially at 4.0% concentration had the most significant impacts. Accurate estimation of the parameters of pest population development is necessary for the development of successful pest management plans^66^. Pesticide concentrations that are either lethal or sublethal can change the biological features of both target and non-target species^67^. The phytocompounds in essential oils contribute to their biological activity. It appears that the LGO nanoencapsulation preserved these phytocompounds and protected them from rapid degradation, increasing the biological activity against treated larvae. According to Peres et al.^68^, the controlled release of active compounds may be the reason for the increased biological activity of encapsulated bioactive agents. According to our findings, food that has previously been treated with nano-encapsulated LGO is not favorable to the growth, development, and reproduction of *S. frugiperda*.

## Conclusions

Lemongrass essential oil was successfully nano-encapsulated using beta-cyclodextrin (β-CD) and gum Arabic (GA) as protective wall material. The GC–MS analysis identified D-limonene, E-Citral, and Z-Citral as the main compounds in LGO. The physical characterization of prepared nano-capsules showed that oil concentration affected the particle size, PDI, zeta potential, and encapsulation efficiency measurements. The particle size of nano-capsules increased by increasing oil concentration, whereas the smallest particle size and PDI value, and the highest encapsulation efficiency were measured for 0.5% oil concentration. When tested against the first larval instar of *S. frugiperda*, both non-formulated and nano-formulated oil showed toxic effects; however, the inclusion of LGO:β-CD/GA led to higher mortality rates than pure LGO. In comparison to LGO treatments, the inclusion of LGO:β-CD/GA at four tested concentrations significantly prolonged the larval and pupal duration and reduced adult longevity and fecundity, thereby having a severe effect on the insect’s development. Additionally, the life table parameters such as intrinsic rate of increase, finite rate of increase, as well as the gross and net reproductive rates of *S. frugiperda* were reduced significantly following LGO:β-CD/GA treatments. These findings will be helpful in the management of *S. frugiperda* using eco-friendly nano-formulations. However, more research would be performed to determine the effectiveness of prepared nano-formulations under different environmental conditions and to investigate their impact on non-target organisms.

## Data Availability

The datasets used in the current study are available from the corresponding author on reasonable request.
